# Clinic of Professor Pancoast

**Published:** 1844-05-04

**Authors:** 


### CLINIC OF PROFESSOR PANCOAST.

Professor Pancoast has been enabled, from the great number of patients contained in the various surgical wards, to exhibit during the course of the session a specimen of almost every class of surgical affection. Of those for which operations have been performed, the following list has been prepared by Dr. Reuben S. Calloway, one of the members of the class.

Rhinoplastic Operation,—re-construction of the nose entire from the skin of the forehead.

Various operations for Strabismus.

Operation for Cataract.

Operation for Synechia posterior.

Formation of Artificial Pupil.

Operation for Encanthis.

Operation for Fistula Lachrymalis.
Operation for Lachrymal Tumour.
Operation for the removal of large tumour from the ear.
Various operations for Phimosis.
Case of Paraphimosis.
Operation for Urinary Fistula.
Various operations for Fistula in Ano.
Various operations for Abscess by the side of the rectum.
Operations for Abscess under the fascia lata.
Various operations for Hydrocele.
Operation for Hydrocele of the Cord.
Operation for cohesion of the fingers, (plastic process.)
Operation for Varicocele. Cirsocele.
Operation for deformity of the toes, (subcutaneous operation.)
Amputation of the Thigh, (oval.)
Amputation of the Leg, (flap.)
Cauterisation of the Urethra and Prostate.

In addition to these were presented a number of cases for the purpose of illustrating surgical pathology and therapeutics,—of which may be mentioned:

Almost every variety of syphilitic affection, from the primary chancre, to the various phases of the disease as shown in the secondary and tertiary forms of the affection.
Various cases of Gonorrhœa, in their simple state, as well as in their complication, with hernia humoralis, stricture, &c.
Various forms of Hernia.
Different forms of Ulcers.
Cases of Psoas and Iliac Abscess.
Cases of diseased knee and elbow joints.
Various cases of Caries and Necrosis.

